# Psychical Impotence

**Published:** 1881-08

**Authors:** S. W. Gross


### Psychical Impotence.

The only case of psychical impotence that I have ever met with is the following: A widower, 52 years of age, was engaged to be married, and, despite the fact that he had erections in the presence of the object of his affections, he was so fearful that he would disgrace himself on the night of his wedding, that he made the experiment with another woman and failed utterly. As a consequence of this unfortunate test, he constantly brooded over his imaginary trouble, for which he sought my opinion. I found that his genital organs and prostatic urethra were perfectly normal, and succeeded in obtaining his confidence by assuring him that I had met with many cases of a similar nature, and that they had always yielded readily to teaspoonful doses of fl. ext. damiana, taken every eight hours, for three days before marriage. As a result of this ruse, he subsequently wrote me that the remedy had acted like a charm.—S. W. GROSS, in *Maryland Medical Journal.*

